# Draft Genome Sequence of Commercial Textile Dye-Decolorizing and -Degrading *Bacillus subtilis* Strain C3 Isolated in India

**DOI:** 10.1128/genomeA.00104-16

**Published:** 2016-03-10

**Authors:** Khushbu Kunadia, Neelam M. Nathani, Vishal Kothari, Rohit J. Kotadia, Charmy R. Kothari, Anjali Joshi, Jalpa K. Rank, Priti R. Faldu, M. Chandra Shekar, Mitkumar J. Viroja, Priyank A. Patel, Divyarajsinh Jadeja, Bhaskar Reddy, Ravindra Pal Singh, Prakash G. Koringa, Chaitanya G. Joshi, Ramesh K. Kothari

**Affiliations:** a Department of Biosciences, Saurashtra University, Rajkot, Gujarat, India; b Department of Animal Biotechnology, Anand Agricultural University, Anand, Gujarat, India; c Department of Biotechnology, Junagadh Agricultural University, Junagadh, Gujarat, India; d Department of Biotechnology, Christ College, Rajkot, Gujarat, India

## Abstract

*Bacillus subtilis* C3, a commercial textile dye-decolorizing and -degrading bacterium, was isolated from the common effluent treatment plant (CEPT) of the Jetpur textile dyeing and printing industrial sector situated in the district of Rajkot, Gujarat, India. Here, we present the annotated 4.18-Mb draft genome sequence of *B. subtilis* C3, providing information about the metabolic pathways involved in decolorization and degradation of several commercial textile azo dyes. Thus, we confirm *B. subtilis* C3 as a potential candidate for bioremediation of textile effluents.

## GENOME ANNOUNCEMENT

Several industries make use of dyes and pigments to color products such as textiles, tannery materials, food, paper and pulp, printing materials, carpet, etc. (1). Approximately 15% to 20% of these synthetic dyes enter the environment through effluents generated during the manufacture and processing operations, and the disposal of these wastes into receiving waters causes damage to the environment (2).

Microbial decolorization and degradation of commercial azo dyes are gaining much importance because these processes are eco-friendly and cost-effective. There are reports on isolation and characterization of bacteria capable of decolorization and degradation of a number of textile azo dyes (3).

*Bacillus subtilis* C3 isolated from a common effluent treatment plant (CEPT) exhibited ~95% to 100% decolorization of various azo dyes during 24 to 48 h of incubation under static culture conditions.

To obtain the genome sequence, shotgun sequencing of the isolate was performed using the ion 316 chip and 400-bp chemistry on an Ion Torrent PGM platform per the manufacturer’s instructions. The sequence reads obtained were assembled using CLC workbench, with default parameters resulting in a draft genome of 4,180,465 bp comprising 226 contigs of >200 bp and *N*_50_ size of 81,136 bp. The average coverage of the assembled contigs was 77.4-fold. The 16S rRNA gene alignment result using BLAST against a 16S rRNA gene database gave >98% identity with the *Bacillus subtilis* subsp. *subtilis* strain 168. *Bacillus* species are reported to be potential organisms for the degradation of textile effluents (4). The gene annotation and screening for RNAs were performed using the Rapid Annotation using Subsystem Technology (RAST) server (5). The genome was demonstrated to have 92 RNA molecules and 43.9% GC content. The draft genome revealed the presence of 4,972 coding sequences (CDS), including genes encoding enzymes involved in azo reduction, thus supporting the potential of the strain as a potent candidate for decolorization and biodegradation of textile dyes and also degradation of benzoate and catechol. Apart from these, the draft genome revealed the strain to possess heavy metal reduction and sulfate assimilation capabilities. Information obtained from the whole-genome sequence of the strain helped reveal genetic aspects of the metabolic pathways, which show it to be a promising candidate in the decolorization and biodegradation of various textile dyes and the bioremediation of sites contaminated with textile effluents. Furthermore, in-depth pathway analysis and biochemical characterization of *B. subtilis* C3 under different conditions will enhance our knowledge of the dye-degrading efficiency of this strain and allow its industrial level utilization in treatment of textile dye contamination.

### Nucleotide sequence accession numbers.

The draft genome sequence has been deposited in DDBJ/EMBL/GenBank under the accession number JYOG00000000. The version described in this paper is the first version, JYOG01000000.
